# Reducing CNOT count in quantum Fourier transform for the linear nearest-neighbor architecture

**DOI:** 10.1038/s41598-023-35625-3

**Published:** 2023-05-27

**Authors:** Byeongyong Park, Doyeol Ahn

**Affiliations:** 1 Department of Electrical and Computer Engineering and Center for Quantum Information Processing, University of Seoul, 163 Seoulsiripdae-ro, Dongdaemun-gu, Seoul, 02504 Republic of Korea; 2 First Quantum Inc., 2F-210, Sparkplus, 180, Bangbae-ro, Seocho-gu, Seoul, 06586 Republic of Korea; 3 Physics Department, Charles E. Schmidt College of Science, Florida Atlantic University, 777 Glades Road, Boca Raton, FL 33431-0991 USA

**Keywords:** Engineering, Mathematics and computing, Physics

## Abstract

Physical limitations of quantum hardware often necessitate nearest-neighbor (NN) architecture. When synthesizing quantum circuits using the basic gate library, which consists of CNOT and single-qubit gates, CNOT gates are required to convert a quantum circuit into one suitable for an NN architecture. In the basic gate library, CNOT gates are considered the primary cost of quantum circuits due to their higher error rates and longer execution times compared to single-qubit gates. In this paper, we propose a new linear NN (LNN) circuit design for quantum Fourier transform (QFT), one of the most versatile subroutines in quantum algorithms. Our LNN QFT circuit has only about 40% of the number of CNOT gates compared to previously known LNN QFT circuits. Subsequently, we input both our QFT circuits and conventional QFT circuits into the Qiskit transpiler to construct QFTs on IBM quantum computers, which necessitate NN architectures. Consequently, our QFT circuits demonstrate a substantial advantage over conventional QFT circuits in terms of the number of CNOT gates. This outcome implies that the proposed LNN QFT circuit design could serve as a novel foundation for developing QFT circuits implemented in quantum hardware that demands NN architecture.

## Introduction

Quantum algorithms are becoming important because of their accelerated processing speed over classical algorithms for solving complex problems^1–5^. However, using quantum algorithms to solve practical problems is difficult because quantum states are very susceptible to noise, which can cause critical errors in the execution of quantum algorithms. In other words, quantum errors caused by noise pose a major obstacle to the realization of quantum algorithms.

The quantum circuit model is a well-known model for quantum computation. In this model, quantum algorithms are represented by quantum circuits composed of qubits and gates. Since noise arises from the evolution of quantum states, gate operations are the major cause of noise. Therefore, quantum circuits should be designed with a minimal number of gates, especially in the noisy intermediate-scale quantum (NISQ) arena^6,7^.

Within the realm of quantum logic synthesis, quantum circuits are broken down into gates derived from a universal gate library. The basic gate library consists of CNOT and single-qubit gates^8,9^. Since CNOT gates are considered the main generators of quantum errors and have a longer execution time compared to single-qubit gates^10^, CNOT gates are expected to dominate the cost of quantum circuits when using the basic gate library.

When considering the cost of a quantum circuit, connectivity between qubits should also be taken into account. This is because physical limitations in quantum hardware may enforce quantum circuits to adopt the nearest-neighbor (NN) architecture^10,11^. The NN architecture means that a qubit in the circuit only interacts with adjacent qubits.

The quantum Fourier transform (QFT) is an essential tool for many quantum algorithms, such as quantum addition^12^, quantum phase estimation (QPE)^13^, quantum amplitude estimation (QAE)^3^, the algorithm for solving linear systems of equations^4^, and Shor’s factoring algorithm^1^, to name a few. Therefore, the cost optimization of QFT would result in the efficiency improvement of these quantum algorithms.

There have been studies aimed at reducing circuit costs of QFT^8,14–22^. Among them are studies related to the number of CNOT gates in QFT, including the following:

1. When constructing an $n$-qubit QFT circuit using the basic gate library, $n(n-1)$ CNOT gates are required, provided that qubit reordering is allowed^8^. Qubit reordering implies that the sequence of qubits can be altered before and after the execution of the circuit.
2. In Ref.^14^, the authors incorporated $n(n-1)/2$ extra SWAP gates to develop an $n$-qubit linear nearest-neighbor (LNN) QFT circuit, which accommodates qubit reordering.
  (i) To synthesize a single SWAP gate using the basic gate library, three CNOT gates are required^8^.
  (ii) Consequently, the total number of CNOT gates required for the $n$-qubit LNN QFT circuit presented in Ref.^14^ is $5n(n-1)/2$.
  (iii) By employing SWAP gates in the construction of LNN QFT circuits, the primary term representing the quantity of CNOT gates increases by a factor of 2.5.
3. Previous research efforts, as documented in case studies, have investigated techniques to minimize the amount of SWAP gates required in the LNN architecture when assembling $n$-qubit LNN QFT circuits^15–18^. These studies aimed to optimize the circuit design and improve overall efficiency.

In this paper, we propose a new n-qubit LNN QFT circuit design that directly utilizes CNOT gates, unlike previous studies^14–18^ that utilized SWAP gates. Our approach offers a significant advantage by synthesizing a more compact QFT circuit using CNOT gates instead of SWAP gates, as the implementation of each SWAP gate requires three CNOT gates. Upon qubit reordering, our $n$-qubit LNN QFT circuit requires ${n}^{2}+n-4$ CNOT gates, which are 40% of those in Ref.^14^ asymptotically. Furthermore, we demonstrate that our circuit design significantly reduces the number of CNOT gates compared to the best-known results for 5- to 10-qubit LNN QFT circuits^17,18^.

In the following analysis, we compare our QFT circuit with the conventional QFT circuit^8^ when used as inputs for the Qiskit transpiler^23^, which is required for implementation on IBM quantum computers that necessitate NN architecture^10^. Our findings confirm that using our QFT circuit as input requires fewer CNOT gates in comparison to the conventional QFT circuits. This evidence indicates that our QFT circuit design could serve as a foundation for synthesizing QFT circuits that are compatible with NN architecture, potentially leading to more efficient implementations.

Furthermore, we present experimental results from implementing the QPE using 3-qubit QFTs on actual quantum hardware, specifically the IBM_Nairobi^10^ and Rigetti Aspen-11^11^ systems. We also illustrate the decomposition of controlled-${R}_{y}$ gates that share a target qubit using our proposed method. This particular circuit is often found in QAE, which is anticipated to supplant classical Monte Carlo integration methods^24,25^. By providing these results, we aim to highlight the practicality and effectiveness of our approach in real-world quantum computing applications.

The remainder of this paper is organized as follows: in the “Background” section, we provide a brief overview of quantum circuits, QFT, QPE, and QAE. The proposed approach section outlines our method for constructing LNN QFT circuits. In the results and discussion section, we present the outcomes of transpilation on IBM quantum computers, display the experimental results of QPE executions on quantum hardware, and illustrate how to convert a circuit of controlled-${R}_{y}$ gates sharing the target qubit into an LNN circuit using our proposed method. We also address the limitations of our study and suggest potential future research directions. Finally, we conclude the paper with a summary of our findings and their implications for the field of quantum computing.

## Background

### Quantum circuit

Quantum circuits consist of qubits and gates. Qubits store a quantum state, a vector in a Hilbert space, and each gate represents a unitary transformation on the Hilbert space. The matrix representations of the gates used in this paper are as follows:

1 $$\begin{gathered} H = \frac{1}{\sqrt 2 }\left( {\begin{array}{*{20}c} 1 & 1 \\ 1 & { - 1} \\ \end{array} } \right), CNOT = \left( {\begin{array}{*{20}c} 1 & 0 & 0 & 0 \\ 0 & 1 & 0 & 0 \\ 0 & 0 & 0 & 1 \\ 0 & 0 & 1 & 0 \\ \end{array} } \right), \hfill \\ R_{n} = \left( {\begin{array}{*{20}c} 1 & 0 \\ 0 & {e^{{\frac{i\pi }{{2^{n - 1} }}}} } \\ \end{array} } \right),R_{z} \left( \theta \right) = \left( {\begin{array}{*{20}c} {e^{{ - \frac{i\theta }{2}}} } & 0 \\ 0 & {e^{{\frac{i\theta }{2}}} } \\ \end{array} } \right),R_{y} \left( \theta \right) = \left( {\begin{array}{*{20}c} {{\text{cos}}\left( {\theta /2} \right)} & {{\text{sin}}\left( {\theta /2} \right)} \\ {{\text{sin}}\left( {\theta /2} \right)} & {{\text{cos}}\left( {\theta /2} \right)} \\ \end{array} } \right), \hfill \\ \begin{array}{*{20}c} { SWAP = \left( {\begin{array}{*{20}c} 1 & 0 & 0 & 0 \\ 0 & 0 & 1 & 0 \\ 0 & 1 & 0 & 0 \\ 0 & 0 & 0 & 1 \\ \end{array} } \right), C\left( {R_{n} } \right) = \left( {\begin{array}{*{20}c} 1 & 0 & 0 & 0 \\ 0 & 1 & 0 & 0 \\ 0 & 0 & 1 & 0 \\ 0 & 0 & 0 & {e^{{\frac{i\pi }{{2^{n - 1} }}}} } \\ \end{array} } \right).} \\ \end{array} \hfill \\ \end{gathered}$$

### Quantum fourier transform

QFT is a quantum version of the discrete Fourier transform. The definition of $n$-qubit QFT and its inverse are as follows:

2 $$\begin{gathered} QFT\left| j \right\rangle = \mathop \sum \limits_{k = 0}^{{2^{n} - 1}} e^{{2\pi ijk/2^{n} }} \left| k \right\rangle , \hfill \\ \begin{array}{*{20}c} {QFT^{ - 1} \left| j \right\rangle = \mathop \sum \limits_{k = 0}^{{2^{n} - 1}} e^{{ - \frac{2\pi ijk}{{2^{n} }}}} \left| k \right\rangle .} \\ \end{array} \hfill \\ \end{gathered}$$

**Figure 1:**
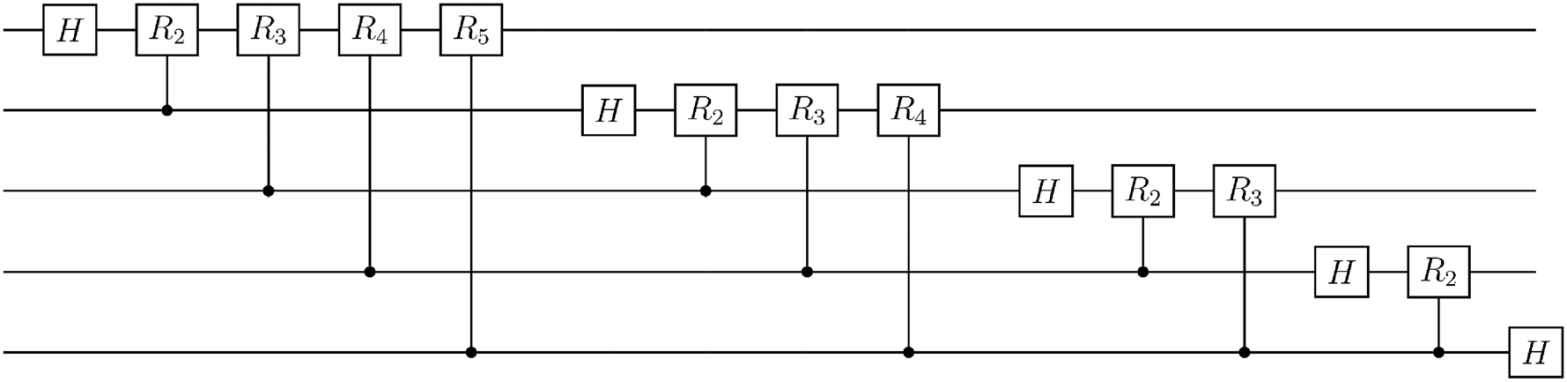
The conventional 5-qubit QFT circuit from Ref.^8^.

The conventional $n$-qubit QFT circuit requires $n(n-1)/2$ $C({R}_{n})$ gates and $n$ Hadamard ($H$) gates if qubit reordering is allowed^8^ (see Fig. 1). Synthesizing a $C({R}_{n})$ gate demands two CNOT and three ${R}_{z}$ gates^26^. Therefore, $n(n-1)$ CNOT gates are required to construct an $n$-qubit QFT circuit. However, if the LNN architecture is required to implement the QFT, the number of CNOT gates is much larger than $n(n-1)$^14–18^.

One of the most important uses of QFT is the QPE algorithm^8,13^. QPE is an algorithm for finding an eigenvector of a unitary operator using QFT. Let a unitary operator, an eigenvalue of the unitary operator, and a corresponding eigenstate be $U$, ${e}^{2\pi i\theta }$, and $\left|u\rangle \right.$, respectively. Then, QPE can find $\theta$ if the state $\left|u\rangle \right.$ is prepared and the controlled-$U$ operators are implemented. The canonical QPE is executed according to the following process: first, prepare the state $\left|{0\rangle }^{\otimes t}\right.\left|u\rangle \right.$, where $t$ is a positive integer related to the precision of QPE. Second, apply $t$ Hadamard ($H$) gates to $\left|{0\rangle }^{\otimes t}\right.$. Third, apply the controlled-${U}^{j}$ operator to the total state, where the controlled-${U}^{j}$ operator transforms $\left|j\rangle \right.\left|u\rangle \right.$ to $\left|j\rangle \right.{U}^{j}\left|u\rangle \right.$, and $\left|j\rangle \right.$ is a computational basis state. Finally, implement the inverse QFT on the first register and measure it. The measurement result gives a number that approximates ${2}^{t}\theta$, which is accurate to $(t-{\mathrm{log}}_{2}(2+1/2\varepsilon ))$ bits with a success probability of at least $(1-\varepsilon )$^8^.

### Quantum amplitude estimation

QAE^3^ is a frequently used subroutine of quantum algorithms. A significant feature of QAE is that it provides a quadratic speed-up compared to the classical Monte Carlo integration^24^.

QAE is an algorithm for finding the amplitude of a state $|{\psi }_{1}\rangle |1\rangle$ in the state $A|{0\rangle }^{\otimes (n+1)}= \sqrt{1-a}|{\psi }_{0}\rangle |0\rangle +\sqrt{a}|{\psi }_{1}\rangle |1\rangle$, where $A$ is a unitary operator. The canonical QAE is the QPE of the Grover operator $Q$. The definition of Q is as follows:

3 $$\begin{array}{c}Q\left(A,\chi \right)\equiv -A{S}_{0}{A}^{-1}{S}_{\chi },\\ {S}_{0}\equiv I-2\left|0\right.\rangle {\left.\langle 0\right|}^{\otimes \left(n+1\right)},\\ {S}_{\chi }\equiv I-2\left|{\psi }_{1}\right.\rangle \left.\langle {\psi }_{1}\right|\otimes \left|1\right.\rangle \left.\langle 1\right|\end{array}$$

Thus, the measurement result correctly converges to $O(1/M)$ with a probability of at least ${8/\pi }^{2}$, where $M$ is the number of qubits representing the measurement result^3^.

Recently, QAEs that do not require the QPE have been proposed^27,28^. They reduce the algorithmic costs compared with the canonical QAE because they do not use additional qubits, controlled operations, nor inverse QFT. However, the QAE without QPE, similar to the canonical QAE, uses the quantum amplitude amplification by the repetitive execution of the Grover operator $Q$. Therefore, reducing the cost of the Grover operator $Q$ is considered a key to efficiently implement QAE.

One of the most frequently appearing subcircuits in the circuit design of a Grover operator $Q$ is the circuit of the serial controlled-${R}_{y}$ gates sharing the target qubit. This is because the serial controlled-${R}_{y}$ gates with single-qubit gates can express the basic approximation form of operator $A$ when QAE is used to implement integration numerically^25^.

The goal of using QAE to implement integration numerically is to find $\sum f(x)$. Then, $A$ and $\theta \left(x\right)$ are defined as follows:

4 $$\begin{array}{c}A{\left|0\right.\rangle }_{n+1}=\sum \sqrt{\left(1-f\left(x\right)\right)}{\left|x\right.\rangle }_{n}\left|0\right.\rangle +\sum \sqrt{f\left(x\right)}{\left|x\right.\rangle }_{n}\left|1\right.\rangle ,\\ \sqrt{f\left(x\right)} \equiv \mathrm{sin}\left(\frac{\theta \left(x\right)}{2}\right),x={\sum }_{k=0}^{n}{x}_{k}{2}^{k}, {x}_{i}= 0\,or\,1.\end{array}$$

Then, $\theta \left(x\right)$ can be written as ${\sum }_{j=0}^{n}{a}_{j}{x}^{j}={a}_{0}+{x}_{0}{\theta }_{0}+{x}_{1}{\theta }_{1}+\dots +{x}_{0}{x}_{1}{\theta }_{01}+\dots$, where each ${\theta }_{k}$ is a linear combination of ${a}_{j}$’s. Therefore, operator $A$ can be approximated to the required precision using a $H$ gate and multi-qubit controlled-${R}_{y}$ gates sharing the target qubit. The basic approximation is the case $n = 1$, which can be synthesized using an $H$ gate, a ${R}_{y}$ gate, and controlled-${R}_{y}$ gates sharing the target qubit. This approximation is useful for solving practical financial problems like risk analysis^25^ or option pricing^29^.

## Proposed approach

**Figure 2:**
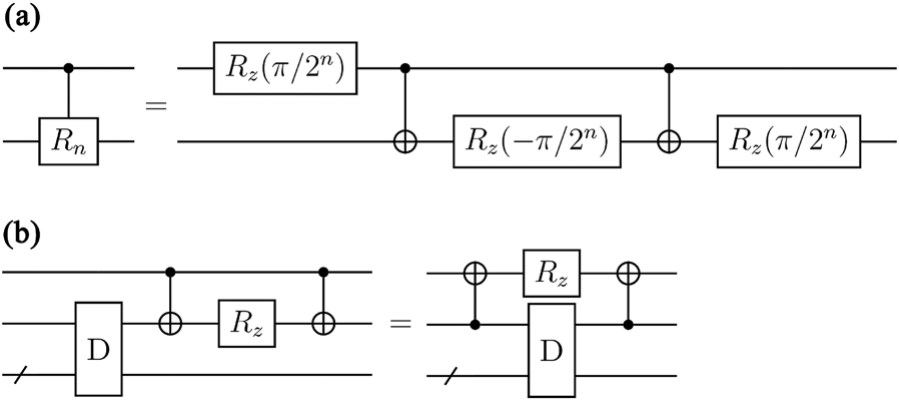
Circuit identities used for decomposing QFT circuits using the basic gate library. (**a**) A circuit identity from Ref.^26^. (**b**) A circuit identity from Ref.^22^. D represents a circuit with a diagonal matrix representation.

**Figure 3:**
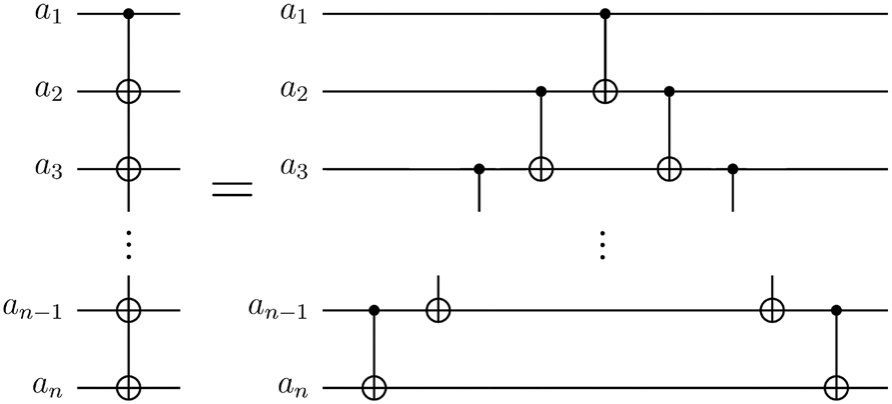
Circuit identity employed to enforce QFT circuits to adopt LNN architecture. This circuit identity holds true for $n\ge 3$.

In this section, we propose a method for constructing an LNN QFT circuit using the basic gate library. This is achieved by applying the circuit identities presented in Figs. 2 and 3. The circuit identity in Fig. 2a is from Ref.^26^, and the one in Fig. 2b is from Ref.^22^. The circuit identity in Fig. 3 is newly introduced in this paper to enforce the QFT circuit to adopt the LNN architecture. The circuit identity in Fig. 3 is proved in Theorem 1.

### Theorem 1

The circuit identity in Fig. 3 holds true for $n\ge 3$.

### *Proof*

It is sufficient to prove that the circuit identity is true when the input state is in an arbitrary computational basis state because quantum mechanics is linear. This can be proved by mathematical induction.

**Figure 4:**
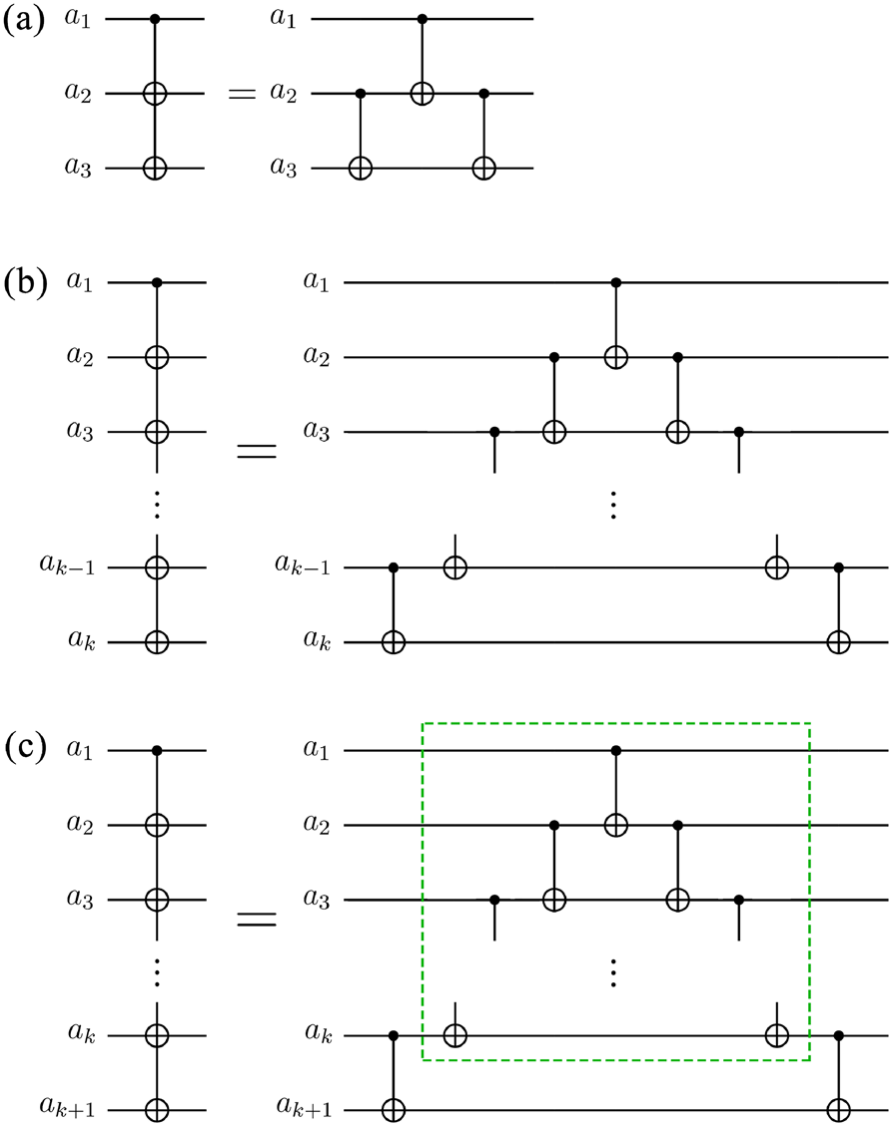
Special cases of the circuit identity in Fig. 3. These cases are utilized in proving Theorem 1. (**a**) The case for $n=3$. (**b**) The case for $n=k$. (**c**) The case for $n=k+1$. The circuit in the dashed green box performs the same operation as the circuit in (**b**).

1. The circuit identity for the case $n=3$ is illustrated in Fig. 4a. Suppose the input state of the circuit in both the left and right circuits of Fig. 4a is in a computational basis state $|{a}_{1}{a}_{2}{a}_{3}\rangle$, where each ${a}_{i}$ is either 0 or 1. In the left circuit of Fig. 4a, the resulting output state is $|{a}_{1}({a}_{1}\oplus {a}_{2})({a}_{1}\oplus {a}_{3})\rangle$. In the right circuit of Fig. 4a, as time progresses, the input state sequentially evolves into the states $|{\psi }_{1}\rangle$, $|{\psi }_{2}\rangle$, and $|{\psi }_{3}\rangle$. The states $|{\psi }_{1}\rangle$, $|{\psi }_{2}\rangle$, and $|{\psi }_{3}\rangle$ are as follows:
  5 $$\begin{array}{c}\left|{\psi }_{1}\right.\rangle =\left|{a}_{1}{a}_{2}\left({a}_{2}\oplus {a}_{3}\right)\right.\rangle \end{array}$$
  6 $$\begin{array}{c}\left|{\psi }_{2}\right.\rangle =\left|{a}_{1}{({a}_{1}\oplus a}_{2})\left({a}_{2}\oplus {a}_{3}\right)\right.\rangle \end{array}$$
  7 $$\begin{array}{c}\left|{\psi }_{3}\right.\rangle =\left|{a}_{1}{({a}_{1}\oplus a}_{2})\left({{a}_{1}\oplus a}_{2}\oplus {a}_{2}\oplus {a}_{3}\right)\right.\rangle =|{a}_{1}({a}_{1}\oplus {a}_{2})({a}_{1}\oplus {a}_{3})\rangle \end{array}$$

Therefore, when $n=3$, the circuit identity is true.

2. Inductive hypothesis: when $n=k$, the circuit identity is true (see Fig. 4b).
3. The circuit identity for the case $n=k+1$ is illustrated in Fig. 4c. Suppose the input state of the circuit in both the left and right circuits of Fig. 4c is in a computational basis state $|{a}_{1}{a}_{2}{a}_{3}\dots {a}_{k}{a}_{k+1}\rangle$, where each ${a}_{i}$ is either 0 or 1. In the left circuit of Fig. 4c, the resulting output state is $|{a}_{1}\left({a}_{1}\oplus {a}_{2}\right)\left({a}_{1}\oplus {a}_{3}\right)\dots \left({a}_{1}\oplus {a}_{k}\right)\left({a}_{1}\oplus {a}_{k+1}\right)\rangle$. In the right circuit of Fig. 4c, as time progresses, the input state sequentially evolves into the states $|{\psi }_{4}\rangle$, $|{\psi }_{5}\rangle$, and $|{\psi }_{6}\rangle$. When the state $|{\psi }_{4}\rangle$ evolves into $|{\psi }_{5}\rangle$, we evaluate the state $|{\psi }_{5}\rangle$ using the inductive hypothesis. The states $|{\psi }_{4}\rangle$, $|{\psi }_{5}\rangle$ and $|{\psi }_{6}\rangle$ are as follows:
  8 $$\begin{array}{c}\left|{\psi }_{4}\right.\rangle =\left|{a}_{1}{a}_{2}{a}_{3}\dots {a}_{k}\left({a}_{k}\oplus {a}_{k+1}\right)\right.\rangle \end{array}$$
  9 $$\begin{array}{c}\left|{\psi }_{5}\right.\rangle =\left|{a}_{1}({{a}_{1}\oplus a}_{2}){({a}_{1}\oplus a}_{3})\dots {({a}_{1}\oplus a}_{k})\left({a}_{k}\oplus {a}_{k+1}\right)\right.\rangle \end{array}$$
  10 $$\begin{array}{c}\left|{\psi }_{6}\right.\rangle =\left|{a}_{1}({{a}_{1}\oplus a}_{2}){({a}_{1}\oplus a}_{3})\dots {({a}_{1}\oplus a}_{k})\left({{a}_{1}\oplus a}_{k}\oplus {a}_{k}\oplus {a}_{k+1}\right)\right.\rangle \\ =\left|{a}_{1}({{a}_{1}\oplus a}_{2}){({a}_{1}\oplus a}_{3})\dots {({a}_{1}\oplus a}_{k})\left({a}_{1}\oplus {a}_{k+1}\right)\right.\rangle \end{array}$$

Therefore, when $n=k+1$, the circuit identity is true.$\square$

**Figure 5:**
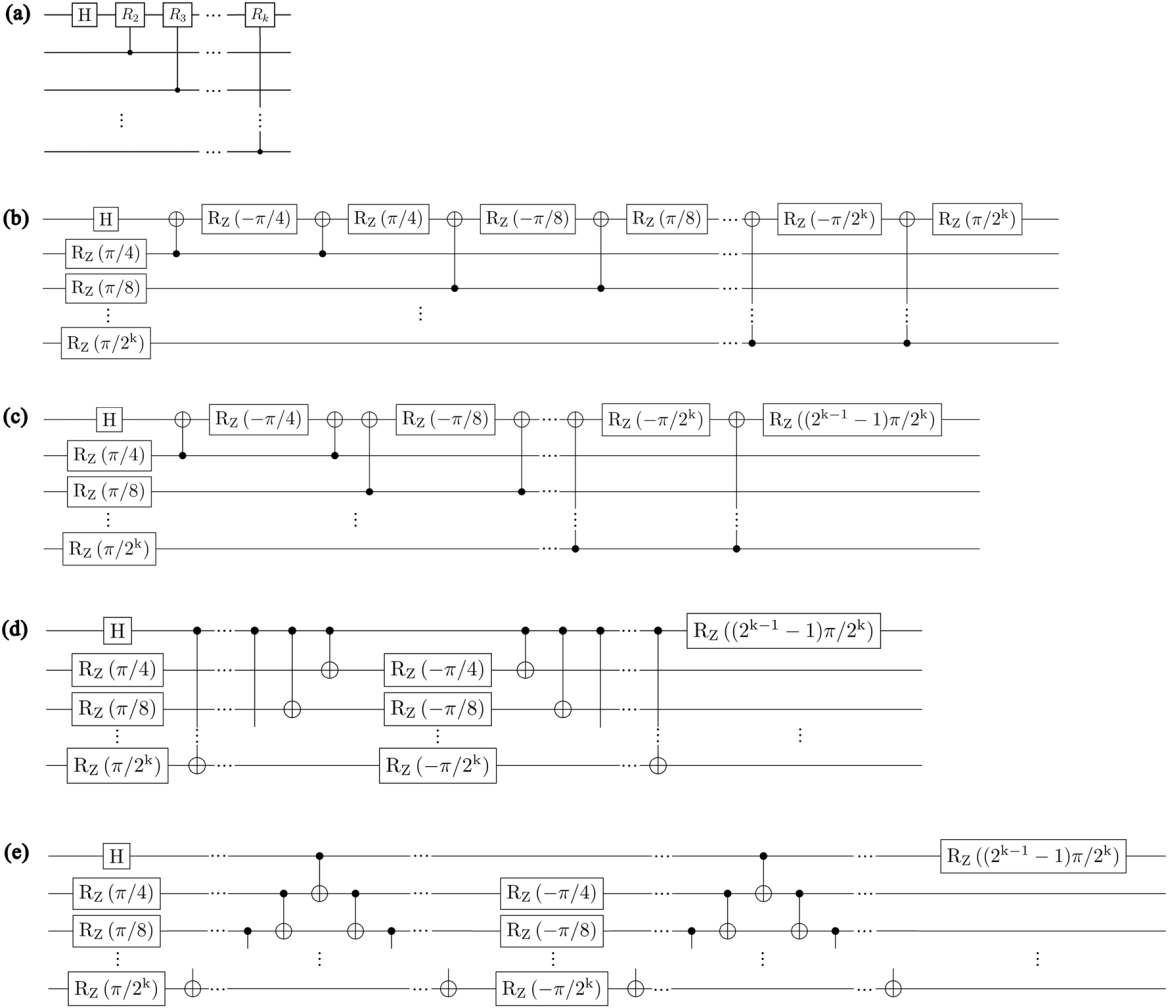
Decomposition of the subcircuit in the QFT circuit. All circuits in this figure perform the same operation. (**a**) A subcircuit of the conventional QFT circuit. (**b**) The result of applying the circuit identity in Fig. 2a to the circuit in (**a**). (**c**) The result of transforming the circuit in (**b**) by utilizing the commutativity of circuits with diagonal matrix representations. (**d**) The result of applying the circuit identity in Fig. 2b to the circuit in (**c**). (**e**) The result of applying the circuit identity in Fig. 3 to the circuit in (**d**).

In the remainder of this section, we present the construction of the LNN QFT circuit. First, we divide the conventional QFT circuit (see Fig. 1) into subcircuits, such as the circuit in Fig. 5a. Next, we decompose the subcircuits using the basic gate library, transform them into circuits for the LNN architecture, and combine them. The process of decomposing the subcircuit in Fig. 5a and transforming it into the circuit for the LNN architecture is as follows:

1. Apply the circuit identity in Fig. 2a to the circuit in Fig. 5a. The circuit identity in Fig. 2a is from Ref.^26^. This step decomposes the circuit in Fig. 5a into the circuit in Fig. 5b.
2. Combine some ${R}_{z}$ gates by using the fact that the circuits represented by diagonal matrices commute with each other. This step transforms the circuit in Fig. 5b into the circuit in Fig. 5c.
3. Repeatedly apply the circuit identity in Fig. 2b, which is from Ref.^22^. This step transforms the circuit in Fig. 5c into the circuit in Fig. 5d.
4. Apply the circuit identity in Fig. 3 to the circuit in Fig. 5d. This step transforms the subcircuit into the circuit for the LNN architecture (see Fig. 5e).

**Figure 6:**
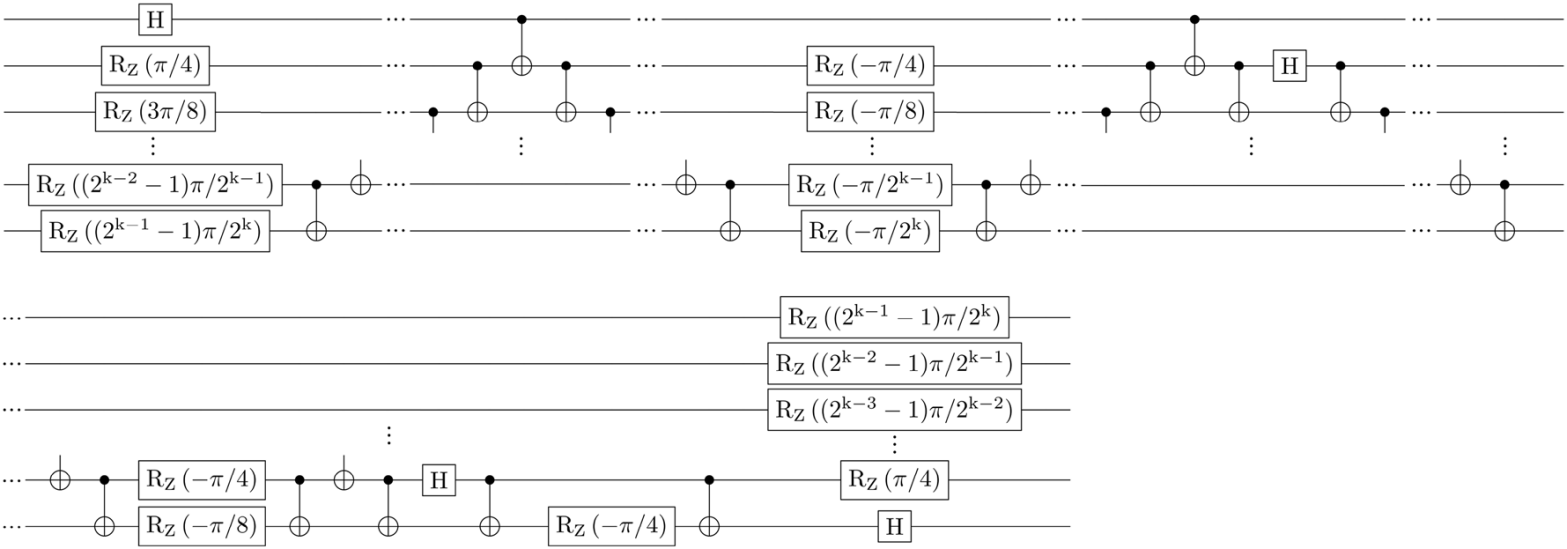
An $n$-qubit LNN QFT circuit with ${n}^{2}+n-4$ CNOT gates.

To construct a QFT circuit, we apply the method above for all the subcircuits, combine them, and cancel out adjacent CNOT gates. Note that this QFT circuit has an LNN architecture (see Fig. 6). Using this method, we can construct an $n$-qubit LNN QFT circuit with ${n}^{2}+n-4$ CNOT gates.

## Results and discussions

### Proposed LNN QFT circuit

Our $n$-qubit LNN QFT circuit requires ${n}^{2}+n-4$ CNOT gates, which is 40% asymptotically when comparing the leading order terms with the previous study in Ref.^14^. Our QFT circuit has the same leading order term of CNOT count ${n}^{2}$, compared to the QFT circuit that does not require an NN architecture. For 5- to 10-qubit QFTs, our results reduce the number of CNOT gates by 16.13%, 20.83%, 30.67%, 43.80%, 47.88%, and 51.89% compared to the best-known results^17,18^. The results and comparison with previous works can be found in Table 1.

**Table 1:** The number of CNOT gates in QFT circuits for LNN architecture.

	Ours	Ref.^14^	Ref.^17^	Ref.^18^	Improvement (%)
n	n^2^ + n − 4	(5/2)(n^2^ − 1)	–	–	~ 60
5	26	50	31	–	~ 16.13
6	38	75	48	–	~ 20.83
7	52	105	105	75	~ 30.67
8	68	140	124	121	~ 43.80
9	86	180	192	165	~ 47.88
10	106	225	240	225	~ 52.89

The first column represents the number of qubits in the QFT circuit, the second column represents our results, the third to the fifth columns represent the results of previous studies^14,17,18^, and the sixth column represents the improvement rate of our circuit compared to the best-known result.

### Transpilation of QFT on IBM quantum computers

**Figure 7:**
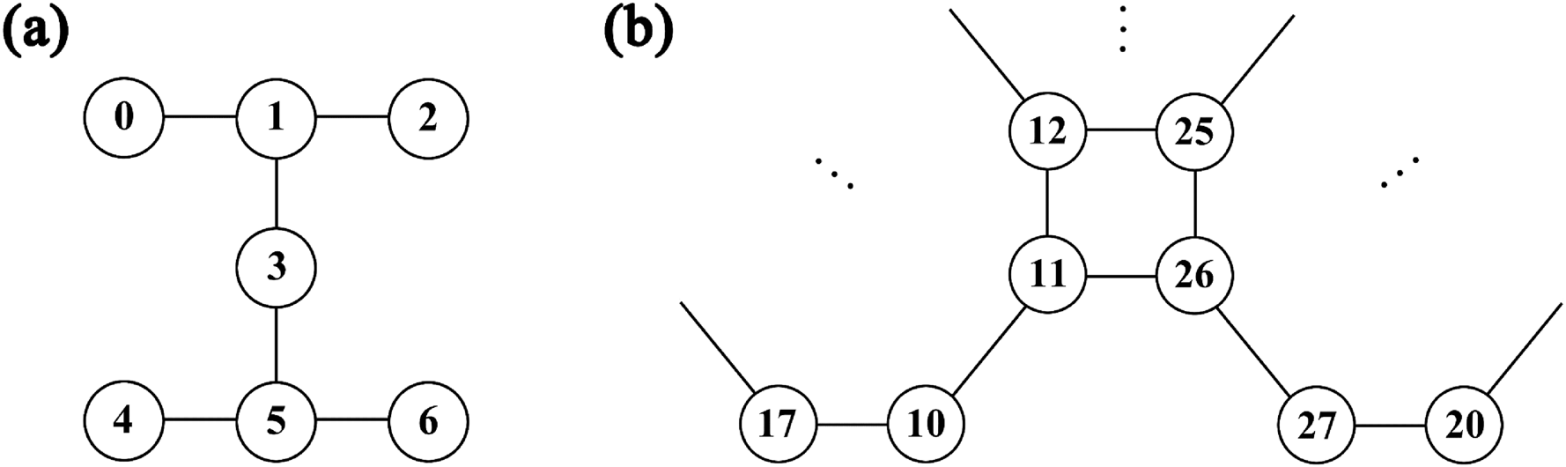
Qubit connectivity of quantum devices (**a**) Circuit diagram of IBM_Nairobi^10^, showing the connectivity of qubits. Qubits labeled 1, 3, 5, and 4 are used to implement QPE. (**b**) Partial circuit diagram of Rigetti-Aspen-11^11^, showing the connectivity of qubits. Qubits labeled 10, 11, 26, and 27 are used to implement QPE.

For real quantum hardware such as IBM quantum computers^10^, the physically implemented circuit must be in a specific NN architecture because qubits are not fully connected. However, the qubits are neither linearly connected (see Fig. 7). Therefore, our QFT circuit for LNN architecture cannot be implemented directly on IBM quantum computers without adjustments for the specific NN architectures.

**Figure 8:**
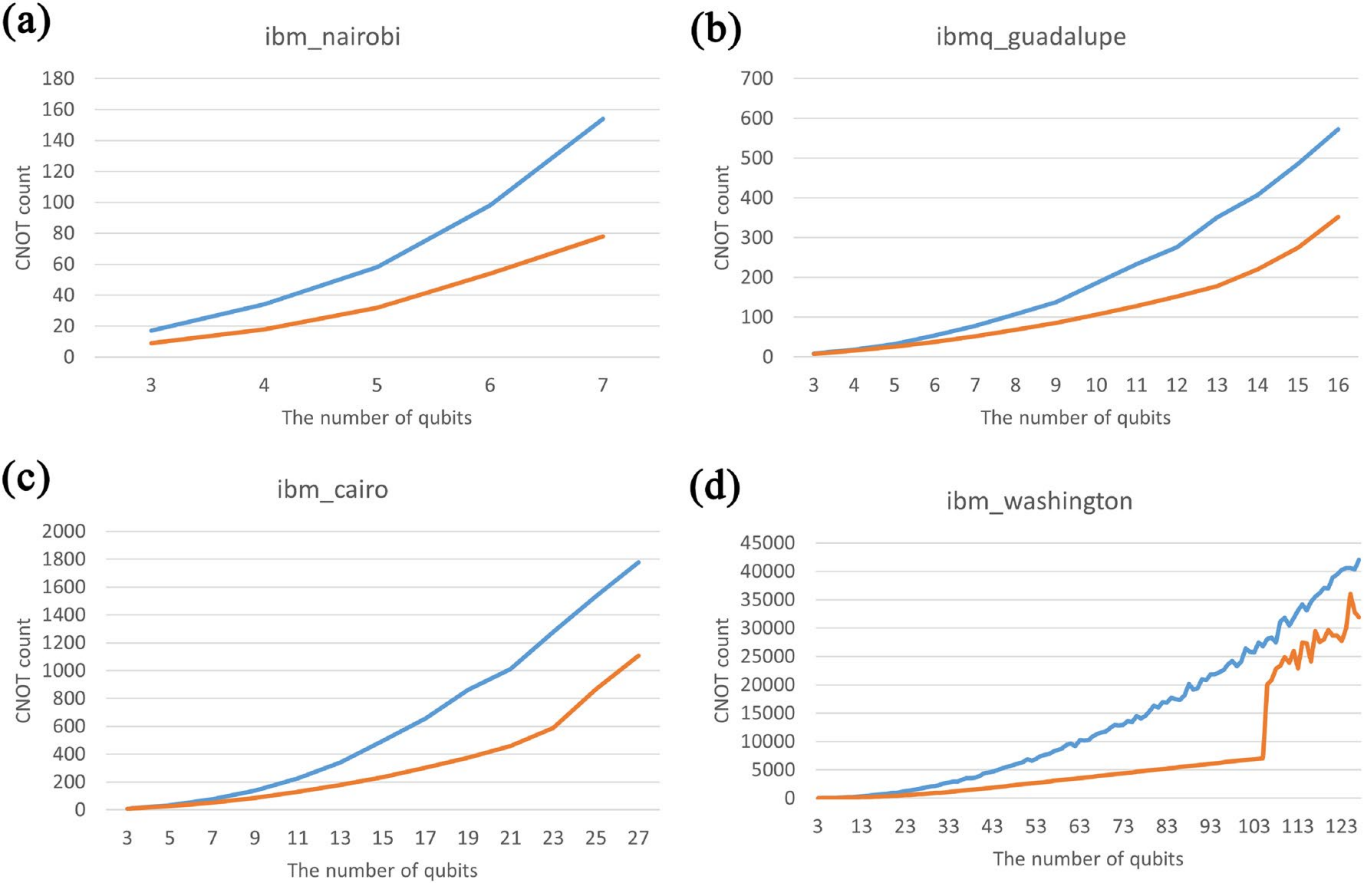
CNOT count for QFT construction on IBM quantum computers. In all figures, the x-axis represents $n$ for an $n$-qubit QFT, and the y-axis represents the required number of CNOT gates for constructing the QFT. The blue lines represent the case using the conventional QFT circuit^8^, while the orange lines represent the case using our QFT circuit. For all cases, our circuit demonstrates an advantage in terms of the number of CNOT gates over the conventional QFT circuit.

Qiskit provides a transpiler^23^ to transform an input circuit into a circuit that satisfies the specific NN condition, which is required in each IBM quantum computer. In this section, we put our QFT circuits and the conventional QFT circuits (such as the circuit in Fig. 1) in the Qiskit transpiler for implementation on IBM quantum computers: (1) IBM_Nairobi, a 7-qubit quantum computer using the Falcon r5.11H processor, (2) IBMQ_Guadalupe, a 16-qubit quantum computer using the Falcon r4P processor, (3) IBM_Cairo, a 27-qubit quantum computer using the Falcon r5.11 processor, and (4) IBM_Washington, a 127-qubit quantum computer using the Eagle r1 processor^10^. We transpiled 3- to 7-qubit QFT on the IBM_Nairobi, 3- to 16-qubit QFT on the IBMQ_Guadalupe, 3- to 27-qubit QFT on the IBMQ_Cairo, and 3- to 127-qubit QFT on the IBM_Washington. Each QFT circuit is transpiled 100 times. Next, we chose the minimal number of CNOT gates required to synthesize the QFT and compared them. As a result, we confirmed that using our QFT circuit as input requires fewer CNOT gates than using the conventional QFT circuit for all cases. The results can be found in Fig. 8.

### Implementation of QFT on actual quantum hardware

We implemented QPE using a 3-qubit QFT on the IBM_Nairobi^10^ and the Rigetti-Aspen-11^11^, a 40-qubit superconducting quantum computer, to compare their performance. The connectivity between qubits used for the implementation of QPE can be found in Fig. 7. QPE is an algorithm for finding an eigenvalue of a unitary operator using a corresponding eigenstate and QFT. A brief explanation of QPE can be found in the “Background” section. In this study, we chose the unitary operator $U$ and the corresponding eigenvector $\left|u\rangle \right.$ as follows:

11 $$\begin{array}{c}U= \left(\begin{array}{cc}1& 0\\ 0& {e}^{2\pi i\theta }\end{array}\right), \left|u\rangle \right.=\left(\begin{array}{c}0\\ 1\end{array}\right)\end{array}$$

We chose $\theta$ as 1/8, 2/8, 3/8, …, and 7/8. The QPE circuits are synthesized using our method. If we use a quantum computer without noise when implementing QPE, we can get the right results with one execution for each $\theta$. However, the quantum computers we used are noisy. Therefore, we implemented QPE 1000 times for each $\theta$ on each quantum computer.

**Figure 9:**
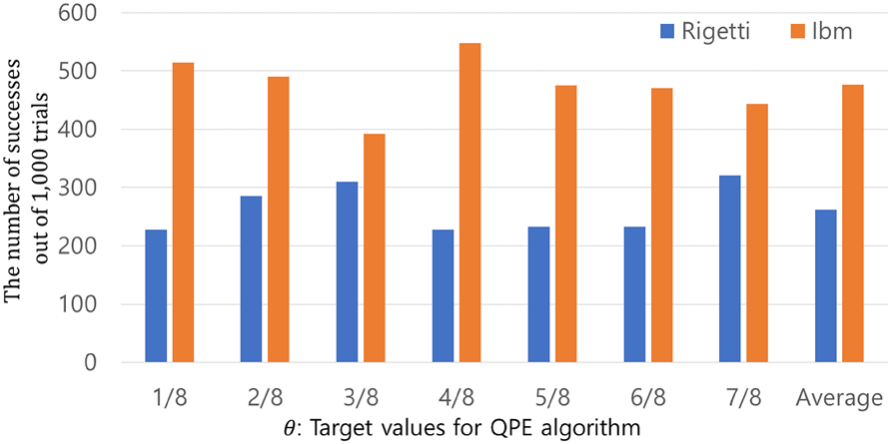
The results and comparison of the implementations of QPEs using 3-qubit QFTs on the Rigetti-Aspen-11 and IBM_Nairobi. The blue and yellow columns represent the results of implementation on Rigetti-Aspen-11 and IBM_Nairobi, respectively. Each QPE was implemented 1000 times for each $\theta$. The x-axis excluding the last one, displays the $\theta$ that QPE aimed to find. The y-axis displays the frequency of the correct $\theta$ being found. The last columns show the averages of the frequency with which the correct answers were obtained.

Utilizing the IBM_Nairobi, we obtained the correct answer by taking a majority vote for all $\theta$. The probability of finding the correct answer was 47.6% on average. We also found the correct answer by using a majority vote for all $\theta$ through the Rigetti-Aspen-11. The probability of finding the correct answer was 26.23% on average. The results and comparison can be found in Fig. 9.

### Applying to QAE circuits

Our proposed method can be utilized to construct other circuits for the LNN architecture. One of the applicable circuits is the circuit of controlled-${R}_{y}$ gates sharing the target qubit. This circuit frequently appears when QAE replaces the Monte Carlo integration^25,27,29^. The explanation of QAE and the reason why controlled-${R}_{y}$ gates frequently appear in QAE circuits can be found in the “Background” section.

The process for transforming the controlled-${R}_{y}$ gates sharing the target qubit into the LNN circuit is as follows:

1. Replace each controlled-${R}_{y}$ gate with a controlled-${R}_{z}$ gate and two ${R}_{x}$ gates using the matrix identity ${R}_{x}\left(-\pi /2\right){{R}_{z}\left(\theta \right)R}_{x}\left(\pi /2\right)={R}_{y}\left(\theta \right)$.
2. Cancel out ${R}_{x}$ gates between controlled-${R}_{z}$ gates.
3. Apply our previously described method for constructing LNN QFT circuits.

### Remarks

The $n$-qubit LNN QFT circuit proposed in this paper requires ${n}^{2}+n-4$ CNOT gates, which is only 40% of the CNOT gates required in the approach presented in Ref.^14^, when considered asymptotically. However, it is important to note that while the LNN QFT circuit in Ref.^14^ exhibits a linear increase in depth with the number of qubits, our LNN QFT circuit experiences a quadratic growth in depth, which might lead to longer execution times. Therefore, future research should focus on minimizing both the number of CNOT gates and the depth of LNN QFT circuits concurrently to further enhance their efficiency.

Moreover, it is essential to recognize that our technique is limited to LNN architectures and does not consider other NN architectures. Given that quantum hardware may not always follow an LNN architecture^10,11^, future work should explore QFT circuit designs for more general NN architectures, such as 2D NN architecture, to ensure broader applicability and utility in the field of quantum computing.

## Conclusion

In this study, we propose a novel LNN $n$-qubit QFT circuit that reduces the number of CNOT gates to approximately 40% of the best-known results. Our QFT circuit does not increase the number of CNOT gates in the leading order term compared to the QFT circuit without an NN architecture. We also demonstrate that transpiling QFT circuits using the proposed design for implementation on IBM quantum computers requires fewer CNOT gates than using conventional QFT circuits. Given these results, our QFT circuit has the potential to replace the conventional QFT circuit as the starting point for QFT circuit optimization in quantum computers that require an NN architecture.

Quantum algorithms that employ QFT may be challenging to implement in the near future because the implementation of QFT requires a large number of quantum gates, which can cause critical errors in executing quantum algorithms. However, QFT is crucial in many essential quantum algorithms, especially those that exhibit exponential speed-up over classical algorithms. Therefore, to fully exploit the advantages of quantum computing, the error rate in implementing QFT should be mitigated. Since our proposed QFT circuit construction reduces the number of CNOT gates, the primary source of errors, our proposal may pave the way for utilizing key quantum algorithms for real-world use cases.

## Data Availability

The data generated during the current study are available from the corresponding author on reasonable request.
